# Effects of shift work on abdominal obesity among 20–39-year-old female nurses: a 5-year retrospective longitudinal study

**DOI:** 10.1186/s40557-016-0148-6

**Published:** 2016-12-05

**Authors:** Gyeong-Jin Lee, Kunhyung Kim, Se-yeong Kim, Jeong-Ho Kim, Chunhui Suh, Byung-Chul Son, Chae-Kwan Lee, Junghye Choi

**Affiliations:** Department of Occupational and Environmental Medicine & Institute of Environmental and Occupational Medicine, Pusan Paik Hospital, Inje University, Busan, Republic of Korea

**Keywords:** Abdominal obesity, Female nurses, Shift work, Waist circumference

## Abstract

**Background:**

This study aimed to investigate the effects of shift work on abdominal obesity among young and middle-aged female nurses during a 5-year retrospective study.

**Methods:**

This retrospective study included female nurses (20–39 years old) who worked at a university hospital in Korea and had available health screening results from 2010–2015. Among 2,611 employees, 934 healthy 20–39-year-old female nurses were identified, and data regarding their demographic information (age and date of employment), waist circumferences (WC), and lifestyle factors (alcohol and exercise) were obtained. Abdominal obesity was defined as a WC of ≥80 cm, based on the World Health Organization’s Asia-West Pacific standard in 2000. The mean WC change from baseline was analyzed using the paired *t* test, and the association between shift work and abdominal obesity was analyzed using the generalized estimating equation.

**Results:**

Compared to all day workers (both age groups), the 20–29-year-old nurses did not exhibit significant changes in WC at each follow-up. However, among the 30–39-year-old nurses, shift workers exhibited a significant change in WC (vs. baseline) during years 4 and 5, compared to day workers. After adjusting for effective confounders and stratifying the participants according to age, the 20–29-year-old nurses exhibited an odds ratio of 3.21 (95 % confidence interval: 1.29–7.98) for shift work-associated obesity, although the odds ratio for the 30–39-year-old nurses was not statistically significant.

**Conclusion:**

In the study population, shift work was associated with a significant change in mean WC among 30–39-year-old nurses, and the shift work-associated risk of abdominal obesity was significant among 20–29-year-old nurses. These results indicate that shift work may influence abdominal obesity differently in 20–29-year-old and 30–39-year-old female nurses.

## Background

Abdominal obesity is a major component of metabolic syndrome, and is considered a preclinical stage of diabetes and cardiovascular disease [1]. Furthermore, the American Medical Association has recognized obesity as a disease that requires greater attention [2]. In this context, shift work is a known risk factor for abdominal obesity, as prolonged exposure to shift work increases the likelihood of having a higher body mass index (BMI) [3, 4]. Other studies have demonstrated that, compared to day workers, shift workers exhibit higher proportions of an increased waist circumference (WC) or abdominal obesity [5, 6]. Moreover, recent systematic reviews have identified an association between shift work and weight gain [7, 8].

Age may be an effect modifier in the association of shift work and obesity [9, 10], and older people may be more vulnerable to shift work-associated risks, as it is difficult for them to readjust their circadian rhythms [11, 12]. A cross-sectional study of male 25–40-year-old workers revealed a higher prevalence of obesity among the shift workers (vs. day workers) [13], and a 1-year cohort study of workers who had recently started shift work revealed significant weight loss, relative to 20–29-year-old day workers [12]. However, these studies did not attempt to provide a hypothesis regarding the underlying reasons for the difference in both studies [12, 13]. Another study analyzed data from the Korean National Health and Nutrition Examination Survey (KNHANES; 2010–2013), and reported that there were no significant changes in weight among 20–29-year-old women [14]. Thus, although previous researchers have identified an effect of shift work on obesity among older age groups, there is insufficient information regarding shift work-associated obesity among younger age groups [6, 12–16]. Therefore, the present study aimed to investigate the effects of shift work on abdominal obesity among young female nurses (20–29 years old vs. 30–39 years old) during a 5-year retrospective study.

## Methods

### Study population

Data were retrospectively extracted from health screening results (2010–2015) that were obtained at a university hospital in Korea. The study included 20–39-year-old female nurses who had been or were currently employed at the hospital. Records from 1,539 female nurses were extracted from the database, although the final eligible population was defined as 934 nurses with more than two health screening results, in order to facilitate beforeafter comparisons. Women who were pregnant or had a chronic illness were excluded. In addition, we were unable to follow-up with female nurses who temporarily resigned or permanently retired after long-term leave (e.g., parental leave or changing jobs) and did not undergo later health screenings. The participants were stratified and categorized into four groups: day workers and shift workers who were 20–29 years old or 30–39 years old.

### Shift work

The work schedule for the nurses was a three-shift rotating system that included day shifts (7 AM to 3 PM), evening shifts (3–10 PM), and night shifts (10 PM to 7 AM). Serial night shifts were usually followed by a day off. The schedules, such as the number of night or evening shifts per month, were similar among the shift workers of all ages. A shift worker was defined as a nurse who had at least four night shifts per month, or >60 night hours per month, during the previous 6 months. Work schedule information was obtained annually through the assistance of the hospital’s administration team, and we assumed that any misclassification of the work schedule would be rare.

### Follow-up

All variables in the present study were measured annually from baseline up to 5 years from baseline, although many participants had unique calendar years for their baseline measurement and subsequent follow-ups. For example, participants who enrolled in 2010 underwent annual follow-ups from 2011 to 2015, and participants who enrolled in 2013 only underwent annual follow-ups in 2014 and 2015.

### Waist circumference and abdominal obesity

WC was measured by trained nurses, although various nurses performed this task during the 5-year study. The WC was measured between the lowest rib and the pelvic iliac crest, with the participant in an upright position and at respiratory expiration. Abdominal obesity was defined as a WC of ≥80 cm, based on the World Health Organization’s Asia-West Pacific standard in 2000 [17]. The baseline WC was defined as the WC from the enrollment year, and the mean changes in WC were calculated based on the WCs from the follow-ups during years 1–5. WC values were measured annually, and the mean changes in WC were calculated for each follow-up evaluation.

### Lifestyle factors

A Korean National Health Insurance Service structured questionnaire [18] was used to obtain personal and family medical histories, and information regarding the participants’ lifestyle factors (smoking, alcohol consumption, and exercise). All lifestyle factors were collected annually from the health screening data, although there were no smokers in the study sample, and we did not consider smoking in our analyses. Drinking behavior was divided into two categories according to the 2014 KNHANES definition [18]: unhealthy drinking (an average of >5 drinks per week or drinking more than twice per week) and healthy drinking (all other cases). Exercise behavior was divided into three categories: light exercise (0 points), moderate exercise (1 point), and intense exercise (2 points). The total weekly amount of exercise was classified as sufficient, basic, or insufficient based on the product of the exercise behavior score and the number of exercise sessions per week. Participants were grouped into the sufficient, basic, or insufficient exercise groups based on total scores of ≥7 points, 4–6 points, and ≤3 points, respectively.

### Statistical analyses

The participants’ general characteristics were analyzed using the chi-square test. The mean change in WC (vs. baseline) was analyzed at each follow-up using a paired *t*-test, and the differences in WC change between day and shift workers were analyzed using an independent *t*-test. Only nurses who worked days or shifts (no change in shift assignment during the follow-up) were included in the univariate analysis of the mean change in WC. To estimate the shift work-associated risk of abdominal obesity, a multivariate analysis was performed using the generalized estimating equation (GEE) to adjust for effective variables (follow-up period and exercise status). We excluded smoking behavior from the multivariate analysis, because there were no smokers in the study population. We also excluded tenure and alcohol consumption from the multivariate analysis, because they were not effective variables in the univariate analyses. The link function for the GEE was binary logistic regression, and the dependent variable was abdominal obesity. An auto-regressive correlation structure was chosen to correct for intra-participant effects. All 934 nurses were included in the multivariate analysis with the GEE, although some of these nurses exhibited changes in assignment (day workers and shift workers) and age during the follow-up. We adjusted the multivariate analysis for effective variables (follow-up period and exercise) that were chosen using backward selection. After stratifying the participants according to age, the odds ratios (ORs) and 95 % confidence intervals (CIs) for abdominal obesity were estimated. All statistical analyses were performed using SPSS software (version 21.0; SPSS Inc., Chicago, IL), and all figures were created using GraphPad Prism (version 6.00; GraphPad Software, La Jolla, CA). Differences were considered statistically significant at a *p*-value of <0.05.

## Results

A total of 934 nurses were enrolled at baseline, and the number of nurses who attended the follow-ups ranged from 897 at the 1-year follow-up to 333 at the 5-year follow-up (Table 1).

**Table 1:** The numbers of nurses who underwent health screening and follow-up each year

	Number (%)		
	Total ^a^	Day workers ^b^	Shift workers ^c^
Health screening year			
2010	632 (100.0)	244 (38.6)	388 (61.4)
2011	667 (100.0)	245 (36.7)	422 (63.3)
2012	678 (100.0)	255 (37.6)	423 (62.4)
2013	655 (100.0)	243 (37.1)	412 (62.9)
2014	669 (100.0)	225 (33.6)	444 (66.4)
2015	540 (100.0)	203 (37.6)	337 (62.4)
Follow-up period			
Baseline ^d^	934 (100.0)	309 (33.1)	625 (66.9)
1-year follow-up	897 (100.0)	296 (33.0)	601 (67.0)
2-year follow-up	716 (100.0)	270 (37.7)	446 (62.3)
3-year follow-up	561 (100.0)	223 (39.8)	338 (60.2)
4-year follow-up	440 (100.0)	177 (40.2)	263 (59.8)
5-year follow-up	333 (100.0)	140 (42.0)	193 (58.0)

^a^ total was defined as all study population (shift workers and day workers)

^b^ day workers were defined as the numbers of nurses who did not include into shift workers

^c^ shift workers were defined as the numbers of nurses who had at least four night shifts per month, or >60 night hours per month during the previous 6 months

^d^ Baseline was defined as the year when each subject was enrolled. Each subject may have different baselines according to her enrollment year

There were 730 nurses who were 20–29 years old at baseline, who included 174 day workers (23.8 %) and 556 shift workers (76.2 %). There were 204 nurses who were 30–39 years old at baseline, who included 135 day workers (66.2 %) and 69 shift workers (33.8 %) (Table 2). The baseline tenures for 20–29-year-old nurses were >1 year, while the baseline tenures for 30–39-year-old nurses were >10 years for both day and shift workers. Most nurses exhibited healthy baseline alcohol consumption and basic or sufficient exercise habits.

**Table 2:** Baseline general characteristics of the study population

	N (%) or mean (SD)					
	20–29 years old			30–39 years old		
	Total	Day workers	Shift workers	Total	Day workers	Shift workers
	730 (100.0)	174 (23.8)	556 (76.2)	204 (100.0)	135 (66.2)	69 (33.8)
Alcohol consumption						
Healthy drinking	702 (96.2)	170 (97.7)	532 (95.7)	199 (97.5)	130 (96.3)	69 (100)
Unhealthy drinking ^a^	28 (3.8)	4 (2.3)	24 (4.3)	5 (2.5)	5 (3.7)	0 (0)
Exercise						
Basic ^b^	239 (32.7)	59 (33.9)	180 (32.4)	83 (40.7)	53 (39.3)	30 (43.5)
Insufficient ^c^	73 (10.0)	18 (10.3)	55 (9.9)	38 (18.6)	27 (20.0)	11 (15.9)
Sufficient ^d^	418 (57.3)	97 (55.7)	321 (57.7)	83 (40.7)	55 (40.7)	28 (40.6)
Tenure (years)	1.3 (2.0)	1.9 (2.5)	1.1 (1.8)	10.4 (4.7)	10.4 (5.1)	10.5 (4.0)
Waist circumference (cm)	68.7 (4.2)	68.4 (4.0)	68.8 (4.3)	70.3 (3.9)	70.1 (3.7)	70.7 (4.2)
Abdominal obesity ^e^	10(1.4)	0 (0)	10 (1.8)	6 (2.9)	3 (2.2)	3 (4.3)

^a^ An average of >5 drinks per week or drinking more than twice per week

^b^ A weekly total exercise score of 4–6 points

^c^ A weekly total exercise score of ≤3 points

^d^ A weekly total exercise score of >7 points

^e^ A waist circumference of ≥80 cm.

* Abbreviations: *SD* standard deviation

The overall rate of loss to follow-up was 24.8 %, and the specific rates for most dependent and independent variables were <30 % (Table 3). The difference in the rate of loss to follow-up was approximately 6 % when we compared the groups with and without abdominal obesity. The group that was lost to follow-up had a slightly larger baseline WC, compared to the follow-up completion group, although the difference was not significantly different (0.5 cm, 95 % CI: – 0.15 to 1.10).

**Table 3:** Multivariate analysis using the generalized estimating equation for abdominal obesity stratified by ages ^a^

	20–29 years old		30–39 years old	
	OR	95% CI	OR	95% CI
Work schedule				
Day work	1.0		1.0	
Shift work	3.21*	**1.29–7.98**	1.46	0.78–2.74
Follow-up period (year)	**1.40†**	**1.23–1.58**	**1.60†**	**1.36–1.87**
Exercise				
Basic	1.0		1.0	
Insufficient	0.42*	**0.18–0.98**	0.33*	**0.14–0.78**
Sufficient	1.04	0.64–1.69	0.77	0.49–1.21

*p < 0.05, †*p* < 0.001

^a^ Adjusted for work schedule, follow-up period and exercise selected by using backward selection

* Abbreviations: *OR* odds ratio, *CI* confidence interval

**Fig. 1:**
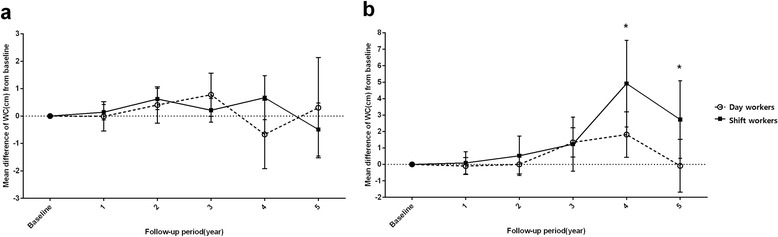
Mean changes in waist circumference among (**a**) 20–29-year-old female nurses. (**b**) 30–39-year-old female nurses. *p <0.05 between day workers and shift workers

Although the mean difference in WC fluctuated during the follow-up period (Fig. 1), different trends in WC changes were observed among the 20-29-year-old and 30-39-year-old nurses. The 20–29-year-old nurses exhibited fluctuating changes, with the day workers exhibiting a non-significant increase in WC at the final follow-up and the shift workers exhibiting a non-significant decrease in WC at the final follow-up. In contrast, the 30–39-year-old nurses exhibited mean changes in WC that started to increase at the second and third follow-ups, peaked at the fourth follow-up (this change from baseline was statistically significant for both day and shift workers), and then decreased at the final follow-up. The 30–39-year-old day workers did not exhibit a significant change at the fifth follow-up, although the 30–39-year-old shift workers exhibited a significant increase in WC (vs. baseline) at the final follow-up.

The multivariate analyses were adjusted for effective variables (work schedule, follow-up period, and exercise). Among the 20–29-year-old nurses, the shift work-related and follow-up period-related ORs for abdominal obesity were 3.21 (95 % CI: 1.29–7.98) and 1.40 (95 % CI: 1.23–1.58), respectively. Among the 30–39-year-old nurses, the shift work-related OR for abdominal obesity was 1.46 (95 % CI: 0.78–2.74), which was not statistically significant, and the follow-up period-related OR was 1.60 (95 % CI: 1.36–1.87). The ORs for the insufficient exercise group remained lower than those for the other exercise groups among both 20–29-year-old and 30–39-year-old nurses.

## Discussion

This study investigated the effects of shift work on abdominal obesity among young female nurses (20–29 years old vs. 30–39 years old) during a 5-year period. The results indicate that 20–29-year-old shift workers had a significant risk of abdominal obesity. Although various studies have reported an association between shift work and obesity, the underlying mechanisms remain unclear [19]. For example, shift work might cause a disruption in circadian rhythms, which could result in lifestyle changes (e.g., eating and exercise habits) and weight gain [20, 21]. In addition, sleep disorders can result in appetite-regulating hormone imbalance (e.g., in leptin and ghrelin) [20, 22]. Furthermore, shift workers might have limited opportunities for physical activity, based on their work schedules and sleep deprivation [23]. Moreover, age may act as an effect modifier in the relationship between shift work and obesity [9, 10], and we hypothesized that age might affect the strength and direction of the association between shift work and abdominal obesity. Unfortunately, there are few studies of people who are <30 years old, and we speculated that this might be related to the limited number of overweight or obese people in that age group, which would limit the ability to statistically evaluate the magnitude of changes in weight or WC.

Young female adults have a higher basal metabolic rate and are more concerned with esthetics and beauty, compared to older women. Therefore, the effects of shift work on abdominal obesity might be confounded by these characteristics, and repeated measures may help estimate the relevant risk. In the present study, we used repeated measures of WC to evaluate the effects of rotating shifts on WC among young female nurses. In the 20–29-year-old group, WC increased during the early study period, but returned to baseline by the fifth follow-up for both shift and day workers. Unfortunately, there is limited research regarding this association among young people, which limits our ability to compare these findings to previous findings. Nevertheless, we propose two explanations for this trend. First, the younger age group may experience a more powerful trend of regression to the mean, compared to the 30–39-year-old nurses. This theory is supported by the strong rebounds that we observed after significant increases in WC, which would imply that the changes were not solely related to the greater basal metabolic rate of the 20–29-year-old group. Thus, the younger age group may perform conscious interventions to control their increasing WC, and this hypothesis is supported by a study of involuntary weight change, which revealed greater involuntary weight gain among the groups that were in their 30s and 40s [14]. A second possible explanation is that the 20–29-year-old age group has a lower prevalence of abdominal obesity, which makes it difficult to set a cut-off value for this group. Thus, a larger sample size may be needed to detect significant differences in subtle WC changes. Furthermore, previous studies have consistently revealed an association between WC change and shift work among 30–39-year-old individuals [12, 24, 25].

Among the 20–29-year-old nurses, the shift work-related OR for abdominal obesity was 3.21 (95 % CI: 1.29–7.98) in the multivariate analysis, which appears to conflict with the absence of a significant change in WC (from baseline) at all follow-ups in the univariate analysis. However, this discrepancy is likely related to the fact that the univariate and multivariate analyses used different dependent variables and the multivariate analyses were adjusted for various confounders. In the present study, abdominal obesity was defined as a WC of ≥80 cm, which reflects the World Health Organization’s Asia-West Pacific standard in 2000 [17]. In contrast, abdominal obesity has been defined as a WC of ≥85 cm for Korean women, although it can be inappropriate to apply a general standard for the female population to an age-specific population, such as in the present study. For example, according to 2014 KNHANES data, 83.5 % of 20–29-year-old women and 53 % of 30–39-year-old women had WC measurements of <80 cm [18]. Furthermore, the present study’s population exhibited a very high proportion of WCs that were <80 cm (20–29-year-olds: 97 %, 30–39-present study’s population exhibited a very high proportion of WCs that were <80 cm (20–29-year-olds: 97 %, 30–39-year-olds: 94 %), which indicates that these individuals had smaller WCs, compared to the general population. Moreover, only approximately 5 % of the included nurses had abdominal obesity (a WC of ≥80 cm), and the mean changes in WC and ORs for abdominal obesity only reflect changes in a small subset of the population. Therefore, we believe that these factors explain the discrepancy between the results of the univariate and multivariate analyses. In addition, the OR for abdominal obesity in the 30–39-year-old group was not statistically significant, although this may be related to the limited number of shift workers in that group.

During the follow-up period, the ORs for increased abdominal obesity over time were 1.40 (95 % CI: 1.23–1.58) among 20–29-year-old nurses and 1.60 (95 % CI: 1.36–1.87) among 30–39-year-old nurses. As all of these ORs are statistically significant, it suggests that the risk of abdominal obesity clearly and steadily increases over time. Moreover, these results are consistent with a previous study’s findings that the prevalence of abdominal obesity increases steadily over time [26].

Only a few participants had unhealthy alcohol consumption habits, and alcohol consumption did not significantly influence abdominal obesity. However, analysis of alcohol consumption may not identify significant effects on WC, as this factor is influenced to a greater extent by food intake, rather than alcohol consumption. Thus, it may be more appropriate to consider the total caloric intake in future studies.

The risk of abdominal obesity among the insufficient exercise group was lower than that among the basic and sufficient exercise groups. We assume that this paradox may be explained by the insufficient exercise group performing less exercise because they were sufficiently slender and did not have to lose weight. Furthermore, 20–39-year-old adults have a higher basal metabolic rate, compared to older individuals, and their weight and WC do not tend to change involuntarily [27]. Moreover, total caloric intake may have a greater effect on abdominal obesity, compared to intense exercise. For example, one study found no statistically significant differences in WC and BMI changes among overweight and obese youth, despite high-intensity interval training [28].

The present study has several limitations. First, the study population does not reflect the general population, because it only included female nurses from a single hospital in a specific region. Second, our data were collected from health screening results and did not include basic information regarding the individual’s obstetric history, socio-economic status, or details regarding their diet and eating habits. We also could not obtain accurate data regarding the nurses’ reasons for retirement and physical workload. However, we assumed that our participants (young female nurses working in the same university hospital) were likely to have similar socioeconomic statuses, and we restricted the study population to only female nurses in order to simplify the analyses and adjustments. Third, our study may include selection bias, as the acute adverse effects of shift work (e.g., sleep disorders and gastrointestinal problems) may contribute to weight loss [29]. Moreover, weight loss-prone individuals may have dropped out of the study before the one-year follow-up because they tend to quit their job more easily. Loss to follow-up may also have biased our findings, although the overall rate of loss to follow-up in the study population was only 24.8 %, and the specific rates for dependent and independent variables were generally <30 %.

The present study also has several strengths. First, we performed a retrospective longitudinal analysis based on data from a 5-year period. Second, we adjusted for unidentified intra- and inter-individual changes using the GEE for statistical and time series analyses. Furthermore, it was possible to specifically identify inter-participant variability, rather than error, based on the use of repeated measures [30]. Third, the relatively large sample size allowed us to analyze the effects of shift work, despite the relatively low prevalence of abdominal obesity among 20–29-year-old women. These factors may explain the difference between our findings and those of previous cross-sectional studies. Third, we were able to evaluate the influence of age by stratifying the participants into 20–29-year-old and 30–39-year-old groups. Thus, we could identify the associations between shift work and increased WC, and also between shift work and risk of abdominal obesity.

## Conclusions

In conclusion, this study revealed that the shift work-related mean change in WC was significant among 30–39-year-old female Korean nurses, and that the shift work-associated risk of abdominal obesity was significant among 20–29-year-old female Korean nurses. These results indicate that shift work may influence abdominal obesity differently among 20–29-year-old and 30–39-year-old female nurses. The mean change in WC among 30–39-year-old female nurses may result from a combination of shift work and a decreased basal metabolic rate, which may be related to the effects of aging. In contrast, shift work had very little effect on the mean change in WC among 20–29-year-old female nurses. This result can be interpreted as indicating that 20–29-year-old female nurses were more resistant to increases in their WC (vs. 30–39-year-old female nurses) because of their higher basal metabolic rate. Despite the high resistance to increasing abdominal obesity among 20–29-year-old female nurses, it is remarkable that shift work may influence their ability to control their weight. Thus, even among a minority of 20–29-year-old female nurses, this study confirmed the existence of a group that is vulnerable to shift work. Therefore, even young female shift workers may require special attention and strategies to reduce their risk of abdominal obesity.

## Abbreviations

CI: Confidence interval
GEE: Generalized estimating equation
KNHANES: Korean National Health and Nutrition Examination Survey
OR: Odds ratio
WC: Waist circumference

## References

[1] Wang XS, Armstrong ME, Cairns BJ, Key TJ, Travis RC (2011). Shift work and chronic disease: the epidemiological evidence. Occup Med (Lond).

[2] Stoner L, Cornwall J (2014). Did the American Medical Association make the correct decision classifying obesity as a disease?. Australas Med J.

[3] van Amelsvoort LG, Schouten EG, Kok FJ (1999). Duration of shiftwork related to body mass index and waist to hip ratio. Int J Obes Relat Metab Disord.

[4] Pan A, Schernhammer ES, Sun Q, Hu FB (2011). Rotating night shift work and risk of type 2 diabetes: two prospective cohort studies in women. PLoS Med.

[5] Mohebbi I, Shateri K, Seyedmohammadzad M (2012). The relationship between working schedule patterns and the markers of the metabolic syndrome: comparison of shift workers with day workers. Int J Occup Med Environ Health.

[6] Lajoie P, Aronson KJ, Day A, Tranmer J (2015). A cross-sectional study of shift work, sleep quality and cardiometabolic risk in female hospital employees. BMJ Open.

[7] Proper KI, van de Langenberg D, Rodenburg W, Vermeulen RC, van der Beek AJ, van Steeg H (2016). The relationship between shift work and metabolic risk factors: a systematic review of longitudinal studies. Am J Prev Med.

[8] van Drongelen A, Boot CR, Merkus SL, Smid T, van der Beek AJ (2011). The effects of shift work on body weight change - a systematic review of longitudinal studies. Scand J Work Environ Health.

[9] Karlsson B, Knutsson A, Lindahl B (2001). Is there an association between shift work and having a metabolic syndrome? Results from a population based study of 27,485 people. Occup Environ Med.

[10] Porta M (2014). A dictionary of epidemiology.

[11] Niedhammer I, Lert F, Marne MJ (1996). Prevalence of overweight and weight gain in relation to night work in a nurses’ cohort. Int J Obes Relat Metab Disord.

[12] van Amelsvoort LG, Schouten EG, Kok FJ (2004). Impact of one year of shift work on cardiovascular disease risk factors. J Occup Environ Med.

[13] Parkes KR (2002). Shift work and age as interactive predictors of body mass index among offshore workers. Scand J Work Environ Health.

[14] Kwon J, Park JW, Park JS, Kim S, Choi H, Lim S. The relationship between night work and involuntary weight change: data from the fifth Korea National Health and Nutrition Examination Survey (KNHANES 2010–2012). Ann Occup Environ Med. 2016; doi: 10.1186/s40557-016-0088-1. 10.1186/s40557-016-0088-1 PMC4731963 26835130

[15] Kim MJ, Son KH, Park HY, Choi DJ, Yoon CH, Lee HY (2013). Association between shift work and obesity among female nurses: Korean Nurses’ Survey. BMC Public Health.

[16] Peplonska B, Bukowska A, Sobala W (2015). Association of rotating night shift work with BMI and abdominal obesity among nurses and midwives. PLoS One.

[17] International Obesity Task Force. The Asia-Pacific perspective: redefining obesity and its treatment. 2000. http://www.wpro.who.int/nutrition/documents/Redefining_obesity/en/. Accessed 9 May 2016.

[18] KNHANES. 2014 Korea national health statistics I, II. 2016. https://knhanes.cdc.go.kr/knhanes/index.do. Accessed 31 Mar 2016.

[19] Amani R, Gill T (2013). Shiftworking, nutrition and obesity: implications for workforce health- a systematic review. Asia Pac J Clin Nutr.

[20] Taheri S, Lin L, Austin D, Young T, Mignot E (2004). Short sleep duration is associated with reduced leptin, elevated ghrelin, and increased body mass index. PLoS Med.

[21] Morselli LL, Guyon A, Spiegel K (2012). Sleep and metabolic function. Pflugers Arch.

[22] Lowden A, Moreno C, Holmback U, Lennernas M, Tucker P (2010). Eating and shift work - effects on habits, metabolism and performance. Scand J Work Environ Health.

[23] Boggild H, Knutsson A (1999). Shift work, risk factors and cardiovascular disease. Scand J Work Environ Health.

[24] Macagnan J, Pattussi MP, Canuto R, Henn RL, Fassa AG, Olinto MT (2012). Impact of nightshift work on overweight and abdominal obesity among workers of a poultry processing plant in southern Brazil. Chronobiol Int.

[25] Antunes Lda C, Jornada MN, Ramalho L, Hidalgo MP (2010). Correlation of shift work and waist circumference, body mass index, chronotype and depressive symptoms. Arq Bras Endocrinol Metabol.

[26] Reis JP, Loria CM, Lewis CE, Powell-Wiley TM, Wei GS, Carr JJ (2013). Association between duration of overall and abdominal obesity beginning in young adulthood and coronary artery calcification in middle age. JAMA.

[27] Manini TM (2010). Energy expenditure and aging. Ageing Res Rev.

[28] Garcia-Hermoso A, Cerrillo-Urbina AJ, Herrera-Valenzuela T, Cristi-Montero C, Saavedra JM, Martinez-Vizcaino V. Is high-intensity interval training more effective on improving cardiometabolic risk and aerobic capacity than other forms of exercise in overweight and obese youth? A meta-analysis. Obes Rev. 2016; doi: 10.1111/obr.12395. 10.1111/obr.12395 26948135

[29] Joseph L, Robert H. Current occupational and environmental medicine. 5th ed. International edition: McGraw-Hill Education; 2014.

[30] Howell DC (2012). Statistical methods for psychology.

